# The effect of somatostatin analogues on postoperative outcomes following pancreatic surgery: A meta-analysis

**DOI:** 10.1371/journal.pone.0188928

**Published:** 2017-12-06

**Authors:** Xianlin Han, Zhiyan Xu, Shaobo Cao, Yupei Zhao, Wenming Wu

**Affiliations:** 1 Department of General Surgery, Peking Union Medical College Hospital, Chinese Academy of Medical Science and Peking Union Medical College, Beijing, China; 2 Department of Vascular Surgery, Wuhan Central Hospital, Tongji Medical College, Huazhong University of science and Technology, Wuhan, China; Georgia Regents University, UNITED STATES

## Abstract

**Background:**

Leakage from the pancreatic stump is a leading cause of morbidity following pancreatic surgery. It is essential to evaluate the effect of somatostatin analogues (SAs) following pancreatic surgery by analyzing all recent clinical trials.

**Data sources:**

We performed a literature search in the Medline, EMBASE, Cochrane Central Register of Controlled Trials and Web of Science databases up to May 29, 2016. Publication bias was assessed with Egger’s test. Study quality was assessed using the Jadad Composite Scale.

**Conclusions:**

Twelve clinical trials involving 1703 patients from Jan 1st, 2000 to May 29th, 2016 were included in the study. With improvements in surgical management and peri-operative patient care, prophylactic use of somatostatin and its analogues reduced the overall incidence of pancreatic fistulas (RR 0.72, 95% CI 0.55–0.94; *p* = 0.02) and decreased the post-operative hospital stay after pancreatic surgery (the weighted mean difference was -1.06, 95% CI-1/88 to -0.23; *p* = 0.01). Other post-operative outcomes did not change significantly with the use of somatostatin analogues.

## Introduction

Pancreatic resection is an essential surgical intervention for patients with pancreatic malignancy and chronic pancreatitis. With the rapid evolution of surgical management in pancreatic diseases encompassing surgical patient selection and refinements in technology, the mortality rate following pancreatic surgery has decreased to 2% at high-volume centers [1].

Leakage from the residual pancreatic stump is a major complication and leading cause of morbidity and mortality following pancreatic surgery. The reported rates range between 10% and 28% [2]. The two most commonly utilized grading systems for postoperative pancreatic fistula, leak and access are the MSKCC (Memorial Sloan-Kettering Cancer Center) and ISGPF (International Study Group on Pancreatic Fistula) grading systems. In this study, we defined clinically important pancreatic fistulas as grade B or grade C by the ISGPF and those showed obvious clinical symptoms [3, 4]. The corrosive nature of the pancreatic secretions, which are frequently compounded by infection, often causes failure of the pancreatic-enteric anastomoses or duct closure. A closed leak from the pancreatic duct may cause erosion of the gastroduodenal artery, resulting in postoperative bleeding, intra-abdominal abscess, bile leakage or pancreatitis, which may require re-operation, resulting in higher mortality rates and expenses and prolonged hospital stay. Therefore, it is highly desirable to reduce the rate of pancreatic fistula formation.

Somatostatin, a 14-amino acid peptide hormone, inhibits pancreatic exocrine secretions by decreasing the volume of pancreatic juice [5]. Although controversy still exists regarding the ability of somatostatin to reduce the rate of pancreatic fistula formation, prophylactic use of synthetic somatostatin analogues (SAs) is commonly observed in clinical practice. Several meta-analyses have been published in recent years that combined data from all past randomized controlled trials (RCTs) to evaluate the efficacy of SAs [6–10]. Given the rapid evolution of technology in surgical methods, such as laparoscopy, robotic-assistance, and peri-operative care, together with uniform criteria for the administration of SAs, the clinical outcomes following pancreatic surgeries have been dramatically improved [11]. Some of these meta-analyses likely introduced selection biases, not including all upgraded clinical trials [10]. We conducted a relatively up-to-date meta-analysis, including 1703 patients, to provide the current best evidence on this topic.

## Materials and methods

### Review strategy

The review process adhered to the PRISMA (Preferred Reporting Items for Systematic Reviews and Meta-Analyses) statement (S1 Checklist) [12]. Relevant studies citing the use of SAs for patients undergoing pancreatic surgery were identified by conducting a search using the PubMed, EMBASE, Cochrane Central Register of Controlled Trials (CENTRAL), and Web of Science databases for studies published up from Jan 2000 until May 2016. The search words include “randomized controlled trials” or “controlled trials”, and “carcinoma” or “cancer” or “neoplasm”, and “surgical procedures” or “pancreatectomy” or “Pancreaticojejunostomy” or “Pancreaticoduodenectomy”, and “somatostatin” or “octreotide” or “lanreotide” or “pasireotide” or “vapreotide”. A comprehensive search of published articles and systemic reviews was performed to ensure all possible studies were included.

### Eligibility criteria

Inclusion and exclusion criteria were pre-defined to avoid selection bias.

#### Inclusion criteria

(1) Research articles only in English; (2) Sample size in trials was greater than 30 patients.

#### Exclusion criteria

(1) Studies with no information regarding the incidence of postoperative pancreatic fistula; (2) Studies that compared two groups that were both administered SAs with different dosages or with different types of SAs; (3) Articles in the form of a case report, review, letter to the editor, editorial or conference abstract.

### Data extraction

All trials were retrieved and reviewed independently by two authors, and data were extracted using a predefined review form (S1 Table). The methodological quality of the studies included in the meta-analysis was assessed using the Jadad composite scale. It evaluates the studies based on perfect randomization, proper blinding, and adequate descriptions of withdrawals and dropouts [13]. Differences in the assessment between authors were reviewed by a third author, and an inter-interviewer reliability analysis was conducted to settle disagreements on the study inclusions.

### Statistical analyses

Regarding the outcomes, data extracted from the original articles were analyzed using the Review Manager 5.3 software (Cochrane Collaboration). For dichotomous outcomes, we applied the risk ratio (RR) with 95% confidence intervals (CI) as the effect size assessment using fixed effects (Mantel-Haenszel) or random effect (Dersimonian and Laird) models [14]. The weighted mean difference (WMD) with 95% CI was calculated for continuous outcomes. The level of significance was set at *p*<0.05. An “intention to treat” basis was chosen to address missing data in different outcomes [15]. We explored heterogeneity by performing the Cochran Q statistic and the *I*^*2*^ test, a *p*-value < 0.1 was considered significant. *I*^*2*^ index was used as an indicator for quantity of heterogeneity [16].

## Results

### Literature search

Our search strategy yielded 2,234 records from the aforementioned four databases. Two reviewers independently reviewed 1,458 abstracts after exclusion of duplications. Case reports, reviews, letters to the editor, editorials and conference abstracts were also excluded during this step. Reasons for exclusion are presented in S1 Fig. Of the 13 potentially appropriate studies [17–29], one was excluded for comparing different administration methods of somatostatin between the experimental and control group [29]. Finally, 12 RCTs were included in the meta-analysis (Table 1).

**Table 1:** Summary of method and characteristics of included studies.

Author, year	Study Design	No. of patients	Dose regimen	Surgical Approach	Definition of pancreatic fistula	Jadad Composite Score
**Sarr et al, 2003**	DB, PC, M	135/140	V, Pr, 600 μg b.d., 7 days	PD, DP, central pancreatectomy	5×↑AMS, >30ml/day at day5	3
**Goulliat et al, 2001**	DB, PC, M	38/37	S, Po, 6 mg/day infusion, 7 days	PD	5×↑AMS, > 50 ml/day at day 10,	5
**Hesse et al, 2005**	R, S	55/50	O, Pr, 100μg t.d.s., 7 days	PD	5×↑AMS, > 50 ml/day at day 3 for>5days, clinical sign	2
**Suc et al, 2004**	S	122/108	O, I, 100 μg t.d.s., 10 days	PD, DP	4×↑AMS, for>3days and clinical definition	3
**Kurumboor et al, 2015**	R, S	55/54	O, Pr, 100mcg t.d.s., 5 days	PD	3×↑AMS, from day 3 ISGPF	2
**Fernandez-Cruz et al, 2013**	R, PC, S	32/30	O, Po, 100μg t.d.s., 10 days	PD	3×↑AMS, from day 3 ISGPF	5
**Kollmar et al, 2008**	DB, PC, S	35/32	O, Pr, 100μg t.d.s., 7 days	PD	3×↑AMS, at day 3 ISGPF	5
**Katsourakis et al, 2010**	R, S	35/32	S, Pr, 3000 μg/day infusion, 7 days	PD, DP	3×↑AMS, at day 3 ISGPF	3
**Anderson et al, 2014**	NRCT, S, Cohort study	79/100	O, I, 300 μg t.d.s., 5 days	PD, DP	3×↑AMS, at day 3 ISGPF	5
**Allen et al, 2014**	DB, R, PC, S	152/148	P, Pr, 900 μg b.d., 7 days	PD, DP	3×↑AMS, at day 3 ISGPF	5
**Yeo et al, 2000**	DB, PC, S	104/107	O, Pr, 250 μg t.d.s., 7 days	PD	3×↑AMS, > 50 ml/day at day 10	4
**Shan et al, 2005**	PC, S	27/27	S, Po, 250 μg/h infusion, 7 days	PD	3×↑AMS, >10ml/day for>7days	3

DB, double blinded; PC, placebo controlled; S, single center; M, multicenter; R, randomized; NRCT, non-randomized controlled trial; V, vapreotide; Pr, pre-operative administration; S, somatostatin; Po, post-operative administration; O, octreotide; b.d., twice daily; t.d.s., three times daily; I, intraoperative administration; P, pasireotide; PD, pancreaticoduodenectomy; DP, distal-pancreatectomy; AMS, amylase;

In total, 1,703 patients were included in the meta-analysis with 853 in the SA group and 850 in the control group. The publication years ranged from 2000 to 2015. Among all of the included studies, eleven were RCTs, and one study was a retrospective nonrandomized study. Seven studies featured pancreaticoduodenectomy (PD) as the only surgical approach, whereas five studies included PD and distal pancreatectomy (DP) as pancreatic surgeries. Three trials used somatostatin, seven used octreotide, one used vapreotide, and one used pasireotide. The definition of a pancreatic fistula was mostly the same among the studies (Table 1). The methodological quality scores of included trials were also estimated at the end of Table 1.

### Primary outcomes

The primary outcomes for this meta-analysis include overall mortality and incidence of pancreatic fistula formation following pancreatic operations.

#### The overall mortality of the SA group was not significantly different from the control group

**Fig 1:**
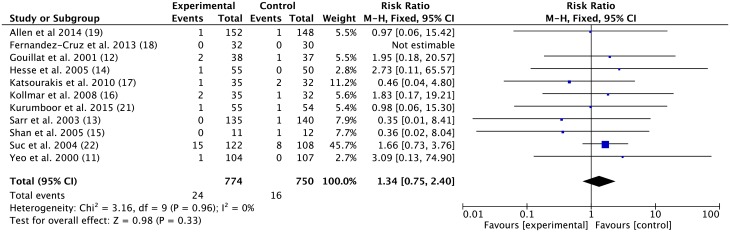
Effect of somatostatin analogues on mortality following pancreatic operations compared with controls. Risk ratios are provided with 95 percent confidence intervals.

Eleven trials noted the mortality rates following pancreatic operations. In total, 69 deaths occurred (40/1524, 2.6%): 24 in the SA group (24/774, 3.1%) and 16 in the control group (16/750, 2.1%). The pooled RR was 1.34 (95% CI 0.75–2.40; *p* = 0.33, fixed-effect model). The heterogeneity was low (Fig 1).

#### The incidence of pancreatic fistula formation of the SA group was significantly different from the control group

**Fig 2:**
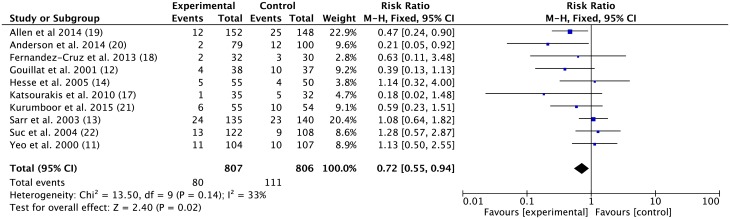
Effect of somatostatin analogues on pancreatic fistula formation following pancreatic operations compared with controls. Risk ratios are provided with 95 percent confidence intervals.

Ten studies were included to analyze the incidence of pancreatic fistulas. The MH RR was 0.72 (95% CI 0.55–0.94; *p* = 0.02, fixed-effect model, Fig 2) with low-to-moderate heterogeneity (*I*^*2*^ = 33%).

No significant publication bias was detected by Egger’s test (*p* = 0.141>0.1, data in S2 Fig). The prophylactic use of SAs reduced the incidence of pancreatic fistula after pancreatic surgery.

#### The incidence of clinical relevant pancreatic fistula formation of the SA group was significantly different from the control group

**Fig 3:**
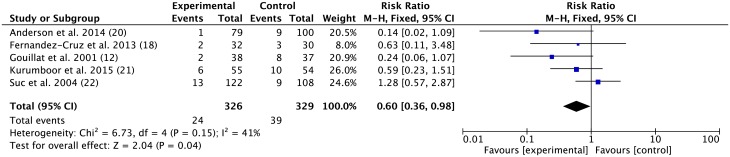
Effect of somatostatin analogues on clinical relevant pancreatic fistula formation following pancreatic operations compared with controls. Risk ratios are provided with 95 percent confidence intervals.

Five studies presenting data of clinical relevant pancreatic fistula were included. The MH RR was 0.60 (95% CI 0.36–0.98; *p* = 0.02, fixed-effect model, Fig 3).

### Secondary outcomes

The secondary outcomes for this meta-analysis were length of postoperative hospital stay, incidence of delayed gastric emptying, intra-abdominal abscesses, postoperative pancreatitis, bleeding and reoperation.

#### The length of postoperative hospital stay of the SA group was significantly different from the control group

**Fig 4:**
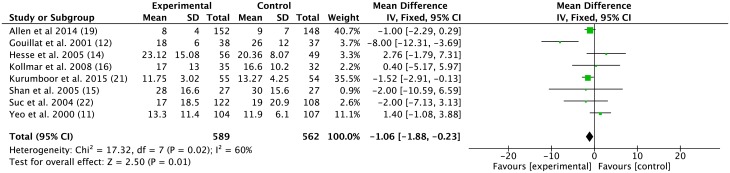
Effect of somatostatin analogues on post-operative hospital stay following pancreatic operations compared with controls. Weight mean differences are provided with 95 percent confidence intervals.

Eight studies noted the mean and standardized deviations of postoperative hospital stays. The weighted mean difference among these studies was -1.06 (95% CI -1.88 to -0.23, *p* = 0.01) with moderate heterogeneity (*I*^*2*^ = 60%) (Fig 4).

There were no significant differences between the SA and control groups regarding the incidence of delayed gastric emptying (RR 0.98, 95% CI 0.62–1.53), intra-abdominal abscesses (RR 0.99, 95% CI 0.66–1.49), postoperative pancreatitis (RR 1.31, 95% CI 0.44–3.88), bleeding (RR 1.07, 95% CI 0.61–1.89), or reoperation (RR 1.31, 95% CI 0.84–2.06).

## Discussion

Despite the fact that the mortality rate of pancreatic surgery has decreased to 2% at high-volume centers [30], pancreatic fistula remains a challenge to surgeons as the main cause of postoperative morbidity and mortality. Various methods from technical to pharmacological aspects, such as surgery methods changing from pancreaticojejunostomy to pancreaticogastrostomy [31] and the use of fibrin sealant [32], have been applied to minimize the incidence of pancreatic anastomotic complications. Somatostatins and its analogues are the most common approaches given their low expense and simple administration, but the use of somatostatins is controversial according to some RCTs and systemic reviews [20, 33–37].

Several systematic reviews and meta-analyses have been performed in recent years that combined data from all published studies. Nevertheless, high heterogeneity may be derived from the long research duration and no consistent definition of postoperative pancreatic fistula. Once a new definition and clinical grading of postoperative pancreatic fistulas are announced by the ISGPF study group and approved by the majority of study centers, researchers will have criteria for comparison of further studies in the future [4]. Given the heterogeneity in trial design and the lack of a consistent definition of postoperative pancreatic fistulas, this efficacy has not been achieved [25]. Besides, heterogeneity results from rapid evolution and refinements in technology and surgical standardization, improvements in patient care and nutritional support during the perioperative period. These changes may also contribute to the different rates of pancreatic fistula among the included studies. Furthermore, definitions of complications vary and were not comparable among previous studies. Taking all into consideration, we finally included clinical trials published from January 2000 to June 2016 to significantly decrease the potential bias and heterogeneity produced by different definitions of complications and surgical management.

Furthermore, Jadad composite scale was applied to evaluate all trials, a high-quality study should have the Jadad score equal to or more than 3 [13]. 83.3%(10/12) of included studies had the Jadad score no less than 2. One of the remaining 2 trials used the clinical grading of pancreatic fistulas by ISGPF. Another study included 3 different surgeons to reduce the potential bias, and low complications rate in both group reflected adequate quality control [20]. Therefore, these 2 trials with Jadad score 2 were also included. The strict enrolling criteria allowed us a relatively high-quality analysis.

Overall, the use of somatostatin did significantly reduce the incidence of pancreatic fistulas probably due to the inclusion of the three latest published trials, which constituted up to 34.5% of the entire cases analyzed. Furthermore, all recent studies suggested that SAs decreased the incidence of pancreatic fistulas after pancreatic surgery. In Allen et al, the high affinity of four (among five) SA subtypes to the somatostatin receptor and longer half-life (up to 11 hours) of pasireotide caused these agents to be more effective at reducing pancreatic exocrine secretions and thus pancreatic fistulas [25]. Kurumboor noted that the categorized post-operative pancreatic fistulas using the ISGPF definition indicated a decreasing trend in clinically significant pancreatic fistulas and claimed that the uniformity of results reporting may cause heterogeneity regarding the effects of prophylactic SAs [27]. Excluding studies prior to 2000 also eliminated heterogeneity regarding surgical management and patient care to some extent. Allpancreatic fistulas were calculated according to published data except two trials [21, 22] that did not clearly describe pancreatic fistulas. Furthermore, we analyzed the incidence of post-operative clinical relevant pancreatic fistulas. Three studies using ISGPF definition and 2 studies using obvious clinical symptoms to define clinical relevant pancreatic fistulas were included. Results showed the risk ratio was smaller than that in the overall pancreatic fistula. This confers a stronger evidence for the protective effect of SAs. Thus, we considered the data reported in the articles as clinically significant. Somatostatin inhibited pancreatic exocrine secretions by decreasing the volume of pancreatic juice [5].

Post-operative hospital stay is an important evaluation index for certain treatment strategies. We observed a statistically reduced post-operative hospital stay in the SA group compared with the control group. A recent study revealed improvements in the gastric-emptying time and mouth-to-caecal emptying time with octreotide in healthy volunteers, and these effects are probably due to the effect of octreotide on gastrointestinal secretion and motility [38]. Patients could potentially obtain better nutrition and faster rehabilitation with early resumption of diet and early discharge from the hospital rather than the beneficial effect of somatostatins in decreasing post-operative pancreatic fistulas.

Nevertheless, prophylactic treatment with SAs did not reduce mortality rates. Given the overall lower mortality rates in all included trials, the study was not originally powered to discriminate on the basis of low mortality. Moreover, given increasing clinical diagnosis experience and the ability to treat serious complications successfully, mortality rates are decreasing further [34].

Regarding postoperative outcomes, the incidence of delayed gastric empty, intra-abdominal abscesses, postoperative pancreatitis, bleeding, and reoperation did not change significantly with the use of SAs. Although research suggested increased gastric emptying due to the downregulation of digestive enzyme secretion by SAs [21], this feature does not significantly affect the prognosis of patients undergoing pancreatic surgeries. Major complications, including bleeding, intra-abdominal abscesses and wound infections, were mainly related to technical pitfalls, which cannot be avoided by prophylactic use of SAs alone. Nevertheless, surgical experience and center size were more essential for the incidence of complications after pancreatic operations [39]. Centers with a high volume of pancreatic surgeries have lower mortality rates compared with lower-volume centers [40], and a policy of centralization may be recommended to decrease the mortality rate [41].

The results of our meta-analysis should be interpreted with caution given several limitations. First, the quality of a meta-analysis depends on its raw data. Although all studies, except one, included in this meta-analysis were prospective controlled trials, considerable heterogeneity still should be considered. Second, six trials utilized placebo control groups, whereas the remaining five trials did not. The inclusion criteria varied among the studies. Half of the trials enrolled patients undergoing only pancreaticoduodenectomy, whereas the other trials recruited all patients undergoing pancreatic resections. Although patients who undergo pancreatic operations for chronic pancreatitis typically have lower rates of post-operative complications, different pancreatic malignancies lead to different outcomes. Furthermore, although the criteria for pancreatic fistulas and the SA formulas were gradually acknowledged, a lack of conformity in the definition of outcome measures and treatment regimens still existed. In eight studies, a fistula was defined as a concentration of drainage with amylase levels three-fold greater than normal as recommended by the ISGFD. Volumes and the number of post-operative days that drainage occurred were considered in four studies. However, the overall heterogeneity of our meta-analysis was relatively reduced compared with previously published systemic reviews.

## Conclusions

Given the improvements in surgical management and peri-operative patient care, the prophylactic use of somatostatin and its analogues appears to reduce the overall incidence of clinical pancreatic fistulas and decreases post-operative hospital stay after pancreatic surgery. Further large, multi-center, prospective RCTs are needed to assess the effects of SAs while stratifying risk factors based on standardized definitions and surgical techniques. Due to the low cost of treatment, the administration of SAs during elective pancreatic surgery is now conventional.

## Supporting information

S1 Checklist PRISMA 2009 checklist. (DOC)

S1 Table Data extracted using predefined fro forma. (DOCX)

S1 Fig Flowchart of the literature search and studies inclusion. (DOC)

S2 Fig Egger’s publication bias plots of pancreatic fistula formation rates. (DOCX)
