# Supplementary material for: The effect of somatostatin analogues on postoperative outcomes following pancreatic surgery: A meta-analysis
Source: PLoS One. 2017 Dec 6;12(12):e0188928. doi: 10.1371/journal.pone.0188928 (PMC5718483; doi:10.1371/journal.pone.0188928)
Supplement: S1 Table — (DOCX) [file pone.0188928.s002.docx]

| Basic characteristics |
| --- |
| first author, year of publication |
| country |
| number of patients in each arm |
| sample size |
| mean age |
| Surgical techniques |
| Types of SAs |
| administration methods of SAs |
| definition of pancreatic fistula |
| Study design |
| Primary outcome |
| mortality |
| incidence of clinically significant PF |
| secondary outcome |
| incidence of intro abdominal abcess |
| delayed gastric emptying |
| bleeding |
| bile leakage |
| pancreatitis |
| reoperation |
| length of postoperative hospital stay |

S1 Table, Data extracted using predefined fro forma
