# Supplementary material for: The effect of somatostatin analogues on postoperative outcomes following pancreatic surgery: A meta-analysis
Source: PLoS One. 2017 Dec 6;12(12):e0188928. doi: 10.1371/journal.pone.0188928 (PMC5718483; doi:10.1371/journal.pone.0188928)
Supplement: S1 Fig — (DOC) [file pone.0188928.s003.doc]

**Screening**

**Included**

**Eligibility**

**Identification**

Records identified through database searching
(n =2234)

Studies excluded for duplications
(n =316)

Abstract screened
(n =1918)

Records screened
(n =266)

Records excluded
(n =253)

Full-text articles assessed for eligibility
(n =13)

Full-text articles excluded, with reasons
(n =1)

Studies included in quantitative synthesis (meta-analysis)
(n =12)

Studies excluded for irrelevant abstract
(n =1652)
