# Supplementary figures and images for: The effect of somatostatin analogues on postoperative outcomes following pancreatic surgery: A meta-analysis
Source: PLoS One. 2017 Dec 6;12(12):e0188928. doi: 10.1371/journal.pone.0188928 (PMC5718483; doi:10.1371/journal.pone.0188928)

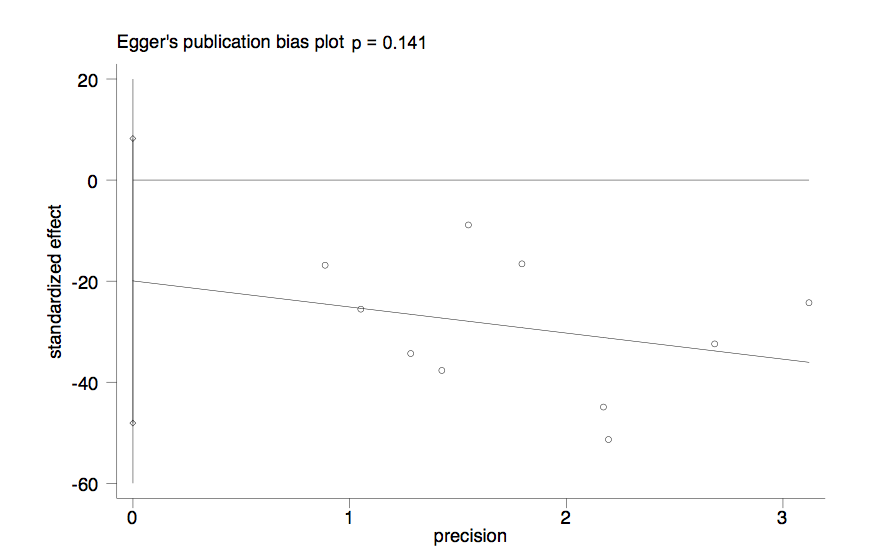

Supplement: S2 Fig — (DOCX) [file pone.0188928.s004.docx]
